# A Recipe for Delirium: Community-Acquired Pneumonia and Sickle Cell Anemia With Moyamoya Disease

**DOI:** 10.7759/cureus.30796

**Published:** 2022-10-28

**Authors:** Franklin Alier, Lyanne Santana, Waiz Wasey, Logan Grubb, Areesha Satti

**Affiliations:** 1 Psychiatry, Southern Illinois University School of Medicine, Springfield, USA; 2 Medical School, University of Medicine and Health Sciences, Springfield, USA; 3 Family and Community Medicine, Southern Illinois University School of Medicine, Springfield, USA; 4 Family Medicine, Southern Illinois University School of Medicine, Springfield, USA; 5 Miscellaneous, Saint James School of Medicine, The Valley, AIA

**Keywords:** community aquired pneumonia, moyamoya disease (mmd), infection induced delirium, and transient ischemic attack (tia), stroke

## Abstract

Moyamoya disease (MMD) is a rare, progressive cerebrovascular disorder that occurs when the major arteries supplying the brain become narrowed or obstructed. Because of this, small and delicate collateral vessels develop to compensate for the decrease in blood flow. Unfortunately, these vessels are insufficient to meet the brain’s metabolic demands. Though initially described in Japan, MMD occurs in a variety of ethnicities around the world. The clinical manifestations of the disease can be devastating, with patients often presenting with symptoms of a stroke or transient ischemic attack. The long history of insults and chronic changes to the brain makes these individuals susceptible to alterations in their mental status. We describe a case of a young African American female with a history of sickle cell anemia (SCA) and undiagnosed MMD who presented to the emergency department with community-acquired pneumonia (CAP). In addition to her medical derangements, she also presented with paranoia, delusional guilt, and refusal to speak.

## Introduction

Getting its name from the Japanese term for “puff of smoke”, Moyamoya disease (MMD) is a rare and irreversible disorder of cerebral vasculature. MMD results from the progressive narrowing of the large intracranial arteries and the subsequent development of small, fragile collateral vessels [1]. These vessels create a smoky appearance on cerebral angiography, hence the name Moyamoya [2]. On the other hand, sickle cell anemia (SCA) is a group of inherited disorders that affect hemoglobin, a protein that transports oxygen throughout the body. In healthy individuals, red blood cells have a disc-like shape, often described as “donuts without a hole.” This shape allows them to maneuver through blood vessels easily. An individual with SCA can develop crescent-shaped red blood cells in response to multiple stressors, such as lack of oxygen, infection, or dehydration. These sickled cells are rigid and unable to bend easily. The rigid cells can block blood flow to the rest of the body. Of note, the most common form of neurologic injury in SCA is silent cerebral infarction [3]. In our case of this 37-year-old female, the combination of SCA and MMD placed her at risk of ischemic stroke, and the long-term consequences of her chronic cerebrovascular disease predisposed her to delirium in the setting of an acute infection.

## Case presentation

A 37-year-old African American woman with a past medical history of SCA and multiple cerebrovascular accidents in childhood was brought to the emergency room by her husband, with complaints of not speaking, eating, or drinking for the past few days. The husband also stated she abruptly stopped taking her medication, hydroxyurea, used to treat her SCA. He stated she had been hypervigilant with her surroundings and paranoid in public lately. He was worried because he had never seen her behave like this before. 

Vitally, the patient was febrile and tachycardic. On gross neurological examination, she was in no acute distress. She would occasionally open her eyes but would not interact with medical staff. The patient could move all of her extremities sporadically but would not follow verbal commands. On motor examination, she could not squeeze the examiner’s hands and would not move her legs for dorsiflexion or pedal push. Motor and sensory examinations were limited due to a lack of patient participation and cooperation. She did not have tremors and her reflexes were 2+ throughout. She was tachypneic on pulmonary examination, and the rest of her physical examination was within normal limits.

Laboratory evaluation showed anemia and leukocytosis (Table 1). Her total and indirect bilirubin levels were elevated. Her liver enzymes, aspartate aminotransferase (AST) and alanine aminotransferase (ALT) levels were mildly elevated as well. All other basic labs on the chem panel were unremarkable.

**Table 1:** Initial laboratory values

LAB	VALUE	REFERENCE RANGE
Hemoglobin (Hb)	7.0 gm/dl	14-18 gm/dl
White blood count (WBC)	13.9 k/mm3	3.4-9.4 k/mm3
Platelets	267 k/mm3	140-410 k/mm3
Aspartate aminotransferase	45 IU/L	13-39 IU/L
Alanine aminotransferase	62 IU/L	7-52 IU/L

**Figure 1:**
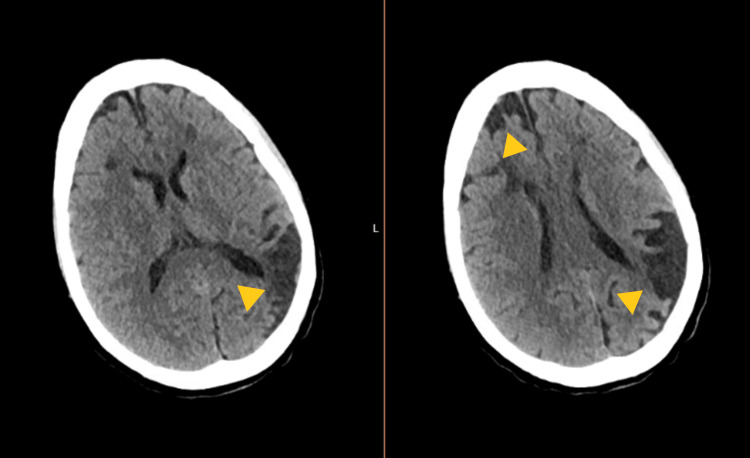
Initial CT scan head showing old infarcts and encephalomalacia

CT angiography of the chest did not show any evidence of a pulmonary embolism. A CT of the abdomen and pelvis showed only hepatomegaly and cholelithiasis. A head CT scan without contrast did not show an acute intracranial abnormality. She did, however, have areas of encephalomalacia suggestive of old infarcts (Figure 1). Her chest X-ray showed interstitial edema, which was concerning for either viral or atypical pneumonia (Figure 3). She was negative for COVID.

**Figure 2:**
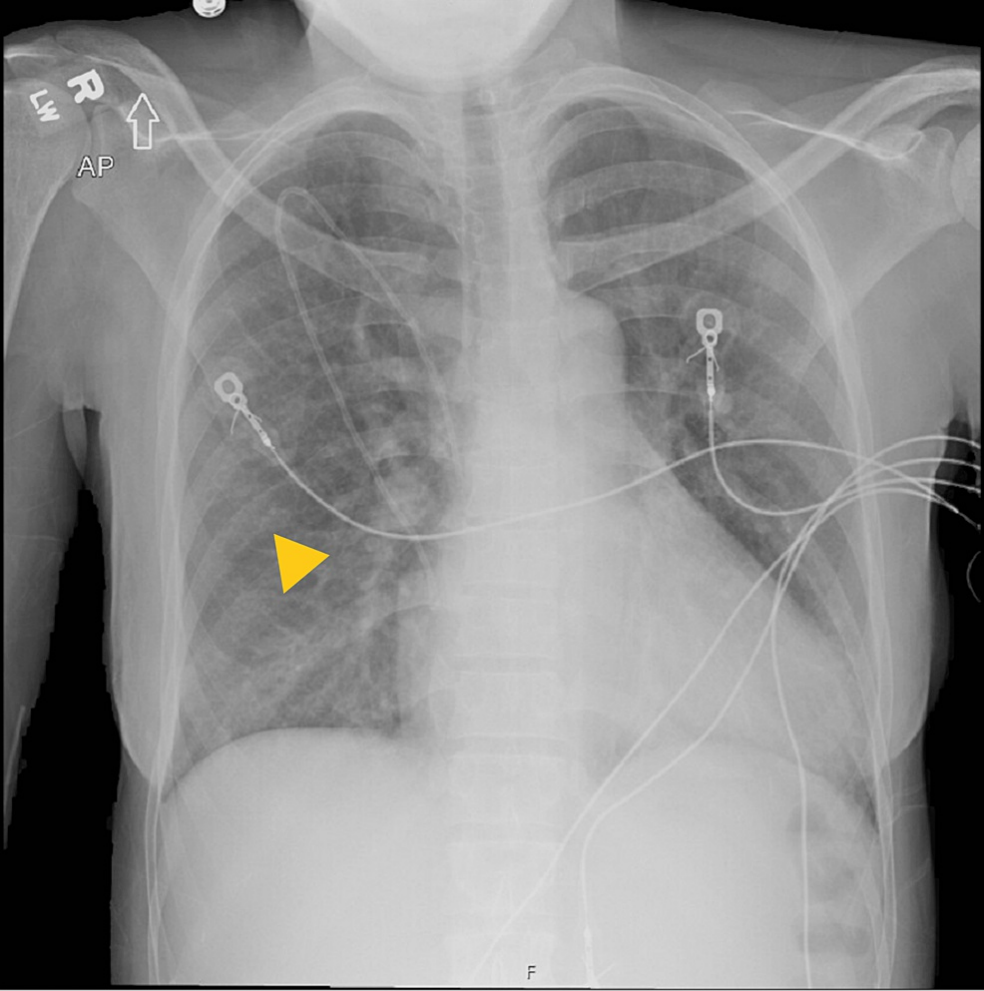
Chest X-ray suggestive of interstitial edema vs viral or atypical pneumonia

The patient was started on community-acquired pneumonia (CAP) treatment with azithromycin and ceftriaxone. Early in her hospitalization, the patient began waking up intermittently and repeating the words “I am sorry”. The delirium was presumed to be secondary to the infection but did not improve with antibiotics even after two days of treatment. Neurology and psychiatry were consulted. They evaluated the patient with MRI, MRA, and MRV imaging studies. A digital subtraction angiography was not done because of unavailability. The patient continued to have episodes of crying frequently and repeatedly stated that she was sorry she hurt her family members.

**Figure 3:**
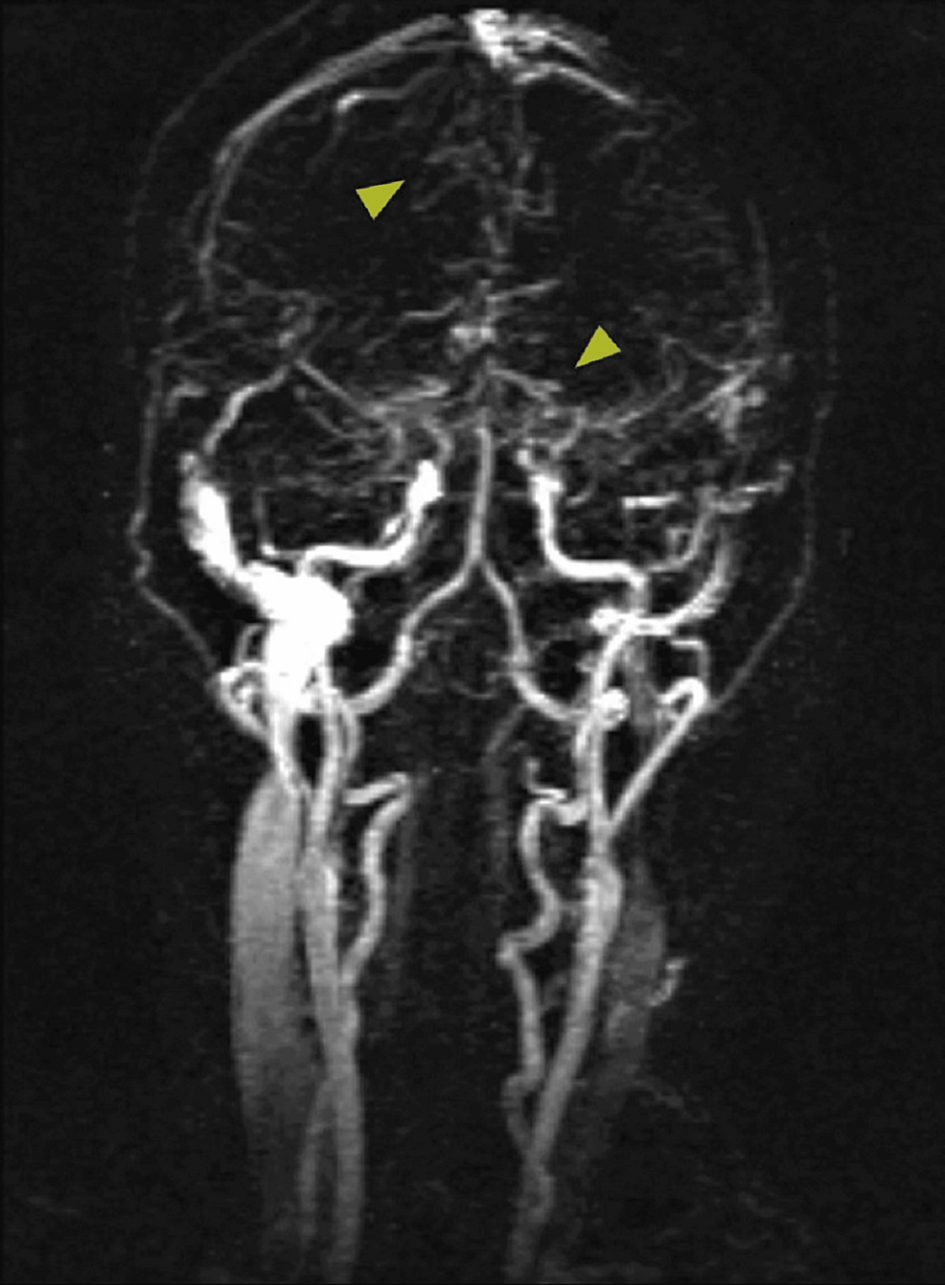
MRA with contrast, showing stenosis of middle, anterior and posterior cervical arteries, giving a "puff of smoke" appearance (Moyamoya)

During a psychiatric evaluation, she mentioned she was feeling extremely depressed. She expressed guilt about her health causing problems for others. She also reported thoughts of wanting to die for the “past few nights.” The husband reported that he had never heard her speak about these thoughts before and was very surprised to hear this. She had a negative prior history of depression. On multiple occasions, the patient was found agitated during her hospitalization and required PRN antipsychotic medications. The results of the MRI and MRA showed evidence of the old ischemic infarcts that most likely occurred during her childhood. The studies also showed arterial narrowing consistent with Moyamoya disease. The distal, cervical segments of the internal carotid arteries were patent. There was, however, significant stenosis of the internal carotid artery termini bilaterally. Multiple collateral arterial structures were seen surrounding the Circle of Willis, which provided further evidence of MMD. Vascular neurology was consulted, who agreed with the MMD as well (Figure 3).

The patient continued to be sad and tearful. Her flow of thought was linear and organized; however, she expressed delusions of guilt. She believed she had infected her entire family with pneumonia. She endorsed passive death wishes but denied suicidal or homicidal ideation. A possibility for paraneoplastic encephalitis was considered by neurology. But thorough evaluations with CT chest, abdomen, and pelvis were negative for any masses suspicious for cancer. The patient was placed on IV steroids and IV immunoglobulins to see if improvement in symptoms, in case it was paraneoplastic encephalitis. There was no drastic improvement in the patient's mentation or mood with the treatment. She scored positive on Confusion Assessment Method (CAM) for delirium.

Psychiatry then placed her on quetiapine for mood disorder. After multiple days of therapy with quetiapine, the patient became more alert and oriented. A repeat CAM was not done. She stated she had no feelings of depression whatsoever and was no longer paranoid. In the outpatient clinic, the patient and her partner continued to state she had no symptoms of delirium on her quetiapine. Since the medicine was making her feel sleepy and tired during the day, it was gradually stopped. She continued to have no delirium or low mood on subsequent follow-up visits. Since the medicine was gradually stopped no acute episodes of irritability were noted. 

## Discussion

The etiology of MMD is currently unknown. Recent genetic studies have identified the RNF213 gene on chromosome 17q25.3 as a significant component that could influence the susceptibility to develop the condition. The mode of inheritance in MMD has not been established either. Several studies have shown a link between familial MMD and chromosomes p26, 6q25, 8q23, and chromosomes 12p12 and 3p24.2. Mutational analysis of RNF213 demonstrated that this mutation dramatically increases the risk of MMD with an odds ratio of 190.8, a 95% confidence interval (71.7-507.9) [4]. The annual incidence of MMD is currently 0.35 to 0.94 per 100,000 population [5]. There is also a female predominance of the disease with a female-to-male ratio of 1.9. A family history of MMD is present in approximately 10-12% of patients.

The most common initial presentation of MMD is an ischemic stroke or transient ischemic attack (TIA). Ischemic stroke or TIA symptoms could present as sudden numbness, weakness, or paralysis. These symptoms will usually be most prominent on one side of the body. A stroke or TIA could cause sudden confusion and difficulty speaking and understanding speech. A sudden loss of vision in one or both eyes could also occur. One of the most frightening consequences of MMD is intracerebral, intraventricular, or subarachnoid hemorrhage. Superimposed delirium could also be seen in patients as a sudden change in alertness and frequent distractibility. Disorganization of speech can occur as well. A delirious patient can also appear drowsy or even semi-comatose in severe cases. Delirium can also be troublesome to diagnose due to its significant overlap with psychiatric symptoms and lack of only being present in certain sections of the older patient population. Hypervigilance, delusions, or even audiovisual hallucinations are common occurrences in delirious patients. Insomnia, depression, anxiety, restlessness, and irritability can also occur. Our patient presented with delirium presentation of MMD.

The gold standard for diagnosis of Moyamoya disease is conventional digital subtraction angiography. This is a fluoroscopic technique performed by inserting a catheter into an artery of the lower extremity and leading it up to the brain's blood vessels. Contrast dye is injected into the cerebral arteries, and X-ray images of the blood vessels are obtained. The stenotic distal internal carotid or proximal arteries of the Circle of Willis and the emblematic, newly formed collateral vessels can be identified by CTA or MRA. 

The mechanisms that lead to arterial stenosis and the formation of small vessel collaterals are known to involve angiogenesis and blood vessel thickening. Some of the factors that are involved in the creation of these new vessels are cytokines, endothelial colony-forming cells, vascular endothelial growth factor (VEGF), and basic fibroblast growth factor (FGF) [6]. In patients with MMD, elevated levels of FGF have been identified within the vascular intima, media, and smooth muscle of blood vessels. Increased levels of FGF have also been found in their cerebrospinal fluid (CSF). Transforming growth factor-beta 1 (TGFB1) has also been identified as an arbitrator of neovascularization [7]. It is believed that it may contribute to the development of MMD. Elevated levels of the hepatocyte growth factor (HGF), another potent agent of angiogenesis, have also been found in the cerebrospinal fluid of patients with MMD [8]. 

Prevention of strokes is an essential component of treating patients with MMD. A recent worldwide survey showed that 31% of respondents had used long-term acetylsalicylic acid (ASA) to prevent strokes in patients diagnosed with MMD. Despite this, there is not much evidence to support the efficacy of long-term ASA therapy in these patients. Recent studies have shown that antiplatelet therapy could not prevent recurrent cerebral infarction in patients with MMD. Surgical revascularization is an effective treatment for preventing ischemic and hemorrhagic strokes in patients with MMD [9]. Compared to conservative measures, surgical revascularization has evidence to support its efficacy. Patients treated with conservative measures only experience annual strokes at a rate between 3.2% and 15%. Comparatively, patients that underwent surgical revascularization showed an annual stroke rate between 0.0% and 1.6%. 

Unfortunately, Moyamoya is a progressive disease. The vascular maladaptation worsens over time due to the extensive intracranial artery occlusions and the development of new collateral vessels. Patients with MMD will often undergo cognitive and neurologic deterioration secondary to progressive vascular changes. MMD patients that remain undiagnosed and untreated can develop significant and progressive neurologic deficits. Outcomes are even less favorable for patients who are active cigarette smokers. Poor outcomes are evident in over 50% of patients. Earlier diagnosis is associated with more favorable outcomes, especially if the disease is diagnosed in childhood. Regarding comorbid MMD and sickle cell anemia, over 40% of patients will experience recurrent cerebrovascular events (CVE) after an initial infarction. The risk of recurrent CVE occurs despite chronic blood transfusions. The risk of recurrent strokes is significantly higher in patients with MMD who have developed collateral blood vessels [10]. Children with SCA are at a significantly increased risk of cerebrovascular accidents. This increased risk was first described in 1923 by Sydenstricker, who had reported seizures and hemiplegia in a five-year-old child [11]. Although the progression of MMD is variable, surgical intervention, especially if it is done prior to the onset of neurological deficits, can improve the prognosis [12].

## Conclusions

This case highlights the unique clinical presentations that can occur in patients with Moyamoya disease and comorbid sickle cell anemia. As the disease is rare, clinicians must uphold a high degree of clinical suspicion for MMD in patients that present with strokes or transient ischemic attacks. Their degree of suspicion for MMD should be even higher in children that present with strokes or TIAs. The chronic cerebrovascular changes, in addition to comorbid SCA, which is commonly associated with MMD, leave these patients predisposed to bouts of delirium, as seen in the context of this 37-year-old patient. Her delirium and seemingly psychiatric symptoms were likely triggered by her SCA, MMD, and ultimately by her underlying community-acquired pneumonia. A reasonably common hospital presentation such as CAP appeared as a case of paranoia, confusion, and delusional guilt in a patient whose MMD had gone undiagnosed and untreated. Although her delirium resolved with a course of quetiapine, her quality of life has been significantly affected by the consequences of her undiagnosed MMD.
